# A Needs-led Framework for Understanding the Impact of Caring for a Family Member With Dementia

**DOI:** 10.1093/geront/gnx148

**Published:** 2017-10-09

**Authors:** Simon Pini, Emma Ingleson, Molly Megson, Linda Clare, Penny Wright, Jan R Oyebode

**Affiliations:** 1 Patient Centred Outcome Research Group, St James’s Institute of Oncology, University of Leeds, West Yorkshire, UK; 2 School of Psychology, Exeter, Devon, UK; 3 Centre for Applied Dementia Studies, University of Bradford, Bradford, West Yorkshire, UK

**Keywords:** Dementia, Caregiving, Qualitative analysis: thematic analysis, Quality of life

## Abstract

**Background and Objectives:**

Approximately half the care for people with dementia is provided by families. It is therefore imperative that research informs ways of maintaining such care. In this study, we propose that a needs-led approach can provide a useful, novel means of conceptualizing the impact of caring on the lives of family carers. Our aim was to develop and present a needs-led framework for understanding how providing care impacts on carers’ fulfilment of needs.

**Design and Methods:**

In this qualitative study, we conducted 42 semistructured interviews with a purposively diverse sample of family carers to generate nuanced contextualized accounts of how caring impacted on carers’ lives. Our inductive thematic analysis focused upon asking: “What need is being impacted here?” in order to generate a needs-led framework for understanding.

**Results:**

Nine themes were widely endorsed. Each completed the sentence: “Being a carer impacts on fulfilling my need to/for….”: Freedom; feel close to my relative; feel in control of my life; be my own person; protect my relative; share/express my thoughts and feelings; take care of myself; feel connected to the people around me; get things done.

**Discussion and Implications:**

These needs echo those from other research areas, with relational needs emerging as particularly central. The needs-led approach offers a perspective that is able to capture both stresses and positive aspects of caregiving. We recommend that clinical interviewing using Socratic questioning to discover human needs that are being impacted by caring would provide a valuable starting point for care planning.

Almost half of dementia care in the United Kingdom is provided by family members at an estimated cost of £26.3 billion (Prince et al., 2014), and in the United States, an estimated 18.1 billion hours of care are provided by family and friends with a value of over $221 billion (Alzheimer’s Association, 2016). Without this care, many people with dementia would have a poorer quality of life and more would have to move into formal care settings (Smits et al., 2007). It is therefore imperative that research addresses issues that can inform the support and maintenance of family-based care post-diagnosis for people with dementia.

The negative impacts on the carer of providing care for a relative with dementia are well documented (Sörensen, Duberstein, Gill, & Pinquart, 2006). Studies have shown that over one in five carers become depressed (Cuijpers, 2005). Carers’ physical health has been shown to be poorer, on average, than that of age-matched noncaregivers and those caring for relatives with other health conditions (Sörensen et al., 2006). A number of models based on stress-appraisal-coping theory have been put forward to describe the paths by which context, stress, appraisal, and mediating or moderating buffers may interact to impact upon the well-being of carers (Pearlin, Mullan, Semple, & Skaff, 1990; Sörensen et al., 2006). Other research recognizes relationship change (Ott, Sanders et al. 2007) or positive aspects of caring (Carbonneau, Caron, & Desrosiers, 2010; Cohen, Colantonio, & Vernich, 2002) as having a major influence on the impact of caring for a relative with dementia.

In this paper, we introduce and apply an alternative approach, the needs-led approach, to conceptualizing the impact on carers of caring for a relative with dementia. This has potential to bring fresh understanding but has not to date been applied to the understanding of family caregiving. In recent years, the needs-led approach has been used to understand the impact of health conditions, providing a valued “patient perspective” on the impact of living with illness (Wilburn, McKenna, Twiss, Kemp, & Campbell, 2013). The needs-led approach can be traced back to the work of Maslow, who proposed that human beings flourish when they are able to fulfil a range of universal human needs (Maslow, 1943). It has been used to understand general human experience (Ryff, 1989) and experience of ageing (Hyde, Wiggins, Higgs, & Blane, 2003), as well as that of living with health conditions.

In the dementia research field, studies of the needs of family carers have tended to focus on care-related needs rather than universal human needs. This is illustrated by a recent systematic review (McCabe, You, & Tatangelo, 2016) in which the authors aimed to review qualitative studies that identified needs from the perspective of family carers. The framework used to capture the findings of the 12 studies reviewed included headings of: information and knowledge needs, informal and formal care needs, other support needs, and self-care needs. These demonstrate that previous studies have predominantly investigated *care* needs rather than exploring *human* needs in the sense proposed by Maslow (1943) or Ryff (1989).

In contrast, Schölzel-Dorenbos, Meeuwsen, & Olde Rikkert, (2010) take the human needs-led approach in their narrative literature review. They propose a hierarchical model, relating conceptual levels of human needs in dementia to those in Maslow’s hierarchy of motivation (Maslow, 1943). Their model is centered on people living with dementia but acknowledges interdependence of people with dementia and carers. It proposes that it is unmet needs for belongingness and love in both partners that results in a sense of carer burden.

In summary, several theoretical models have been proposed to capture the impact of caring for a relative with dementia. Given the evidence that the effects of caring for a relative with dementia are wide-ranging, the needs-led approach could provide a useful unifying framework and inform effective interventions to support carers to continue in their role without deleterious effects on their well-being. In this paper, we report on the use of the needs-led approach to develop an inductive understanding of how providing care to a relative with dementia affects carers’ ability to fulfil their own human needs. Our report describes the initial inductive phase of an ongoing research study to develop a needs-led measure of quality of life for carers of people living with dementia (Wright et al., 2017).

## Design and Methods

This cross-sectional qualitative study employed semistructured interviews, gathered from a purposively diverse sample of dementia carers, to generate nuanced contextualized accounts of the impact of caring on fulfilment of needs. Data were analyzed using inductive thematic analysis (Braun & Clarke, 2006).

### Participants

Primary carers supporting a partner, family member, friend, or neighbor who had a diagnosis of dementia and was living in the community were eligible to take part if they were over 16 years old, able to understand English, and had capacity to consent. We sought to include variation on a range of dimensions which have been shown to influence carers’ experiences (gender, age, ethnicity, kinship, coresidence, rural/urban) (Pearlin et al., 1990). To achieve this, we used a sampling frame with the aim of interviewing men and women with a range of relationships to the person being cared for, from rural and urban areas; including people from a range of educational backgrounds; of varying ages; and of white British, South Asian, and black ethnicities. We estimated that a purposively recruited sample of 48 would give adequate variation in all these areas.

### Procedures

Ethical approval was granted in November 2015 by a UK National Health Service ethics committee. Carers were identified via two-third sector organizations (Carers Leeds and the Alzheimer’s Society), an NHS organization (Bradford District Care NHS Foundation Trust), and by participants identifying other carers known to them. The research team contacted those referred to check eligibility, provide information, answer questions, arrange interviews, and obtain written informed consent.

Interviews were audio recorded and followed a semistructured schedule devised by the research team in collaboration with a carer advisory panel (Supplementary File 1). Open questions were used to explore carers’ experiences, thoughts, and feelings related to challenging and rewarding experiences and events. Socratic questioning was employed to probe implicit assumptions, causes, and implications (Paul & Elder, 2007), through asking carers to reflect on why particular experiences of care made them feel or think as they did. In this way, we aimed to uncover underlying needs that were impacted upon by the carer experience.

### Analysis

Interviews were anonymized at the point of transcription and thematically analyzed (Braun & Clarke, 2006). Data were managed in NVIVO 10. Data collection, analysis, and reflection were interleaved, enabling areas of interest emerging from earlier analysis to be probed in greater depth in subsequent interviews.

In line with the needs-led approach, all segments of text related to positive and negative impact of caring on the carer were *extracted* (McKenna & Doward, 2004). Extracts were inductively *coded* to capture the nature of the impact, returning to the interview context to enhance understanding and using constant comparison to identify similarities and differences. The research team paid constant attention to ensuring the analysis was grounded in the interviews. Socratic questioning continued into analysis, with the research team always asking: “What need is being impacted here?” The interviewers (SP, EI) mapped out their initial thoughts about the interview content, followed by the researchers (JO, SP, EI, EPW) individually reading four transcripts and discussing the content together. This resulted in an initial thematic framework. The research team then individually used this to inform coding of four further transcripts before reflecting together on the framework and developing it further. This process was repeated until the team were satisfied that they had captured the impact of caring on the fulfilment of needs in a way that did justice to participants’ accounts. To gain feedback regarding plausibility and coherence, the emergent themes were presented to health and care professionals, two carer advisors who were not participants in the study and the wider research group.

## Results

### Participants

We were able to make contact with 61/67 carers referred. Two declined participation due to changes in their partner’s health and 19 had attributes already adequately sampled (e.g., white urban-based wives or daughters). Forty-two carers were therefore interviewed (41 at home, 1 at work), with interviews lasting on average 77 min (range 23–150). Despite extending the recruitment period, it proved difficult to recruit as many male South Asian, young (<35 years), and daughter-in-law carers as we would have liked, and we therefore stopped recruitment at 42 participants. However, the only gender-by-ethnicity combination not represented was that of male South Asian carers, and overall, the purposive sampling strategy led to good variation (Table 1).

**Table 1.** Participant Demographics (*n* = 42)

Gender	Female	28	Relationship	Wife	12
	Male	14		Husband	10
				Daughter	14
				Son	4
				Daughter-in-law	1
				Granddaughter	1
Ethnicity	White	33	Time caregiving (months)	Mean	54
	Asian	4		Range	5–180
	Black	4			
				*SD* (N-1)	41
	Other	1			
Age	Mean	62	Coresidence	Living with relative with dementia	33
	Range	31–83			
				Not coresident with relative with dementia	9
Employment	Retired	22	Specific diagnosis of cared-for relative	Alzheimer’s disease	18
	Employed FT	3		Vascular dementia	13
	Employed PT	6		Other	9
	Full-time carer	6		Not known	2
	Unemployed	5			
Carer self-rated health status	Excellent	3	Time person can be left alone	A day and night	6
	Very good	10		A whole day	1
	Good	14		Up to half a day	16
	Fair	11		Up to 1 hour	7
	Poor	4			
				Not at all	12
Education beyond 18 years	Yes	21	Living location	Rural	13
	No	21		Urban	29

### Thematic Outcomes

We derived nine themes, each with excellent reach across the sample, all of which completed the sentence: “Being a carer impacts on fulfilling my need to/for…” (Table 2). The second column of Table 2 displays the number and percentage of carers who had at least one extract coded within a need, demonstrating the reach of themes across the sample.

**Table 2.** Nine Human Needs Impacted by Caring for a Relative with Dementia

Being a carer impacts on fulfilling my need to/for…	Number (percentage) of sample with at least one extract coded to the need
Feel close to my relative	42 (100%)
Feel in control	42 (100%)
Freedom	42 (100%)
Feel connected to the people around me	42 (100%)
Protect my relative	40 (95%)
Take care of myself	40 (95%)
Be my own person	38 (90%)
Share/express my thoughts and feelings	36 (86%)
Get things done	28 (67%)

Each need and how its fulfilment was affected by caring for somebody with dementia is described below. We have ordered the themes by our subjective sense of the centrality of their presence in the carers’ accounts which is also reflected to some extent by their reach as indicated in Table 2.

#### Feel Close to My Relative

All carers wanted to continue relating reciprocally and closely to the relative they cared for. Spouses in particular expressed a strong wish to communicate with and understand the person and provide them with a good quality of life, often feeling their close relationship meant they were the only person who could do this. In addition, the majority of carers felt they had a duty to care for their relative because of the love they shared and the commitment they had made (e.g., through marriage vows).

Dementia eroded the cared-for person’s ability to recall recent and more distant shared moments. In the face of this, all carers felt an increasing distance as they were no longer able to share memories or generate future plans together. Any inability to share a narrative about their lives was a challenge to closeness, and it could be distressing for the carer when their relative did not remember positive shared experiences.

These days [she] can’t even say husband because she doesn’t remember the word, that follows on from not being able to remember my name so didn’t know my name, didn’t know our relationship was different than man and woman, it was husband and wife. (Alfred).

All carers said it was upsetting to see the person they cared for changing, with many saying their relative was no longer the same person they had known before dementia developed. However, this could fluctuate, and some found it upsetting when their relative’s previous personality was fleetingly expressed: “You’ve got to find those little glimmers, those little moments where you get the person back just briefly. Those little glimmers, and you just think, ‘Oh right, yes, that’s how it was. . . . .It’s bittersweet, isn’t it?’” (Abbie)

Dementia can impact on the person’s ability to empathize with others, but carers also described having difficulty understanding the world of the person they cared for. Many carers said they could no longer imagine what their relative’s world was like or how their relative experienced day-to-day life. This increasing inability to empathize with each other seemed to contribute to feelings of interpersonal distance and grief.

You know, I wonder about his inner life. What is that life for him? It’s kind of, you’re so busy getting on with the day-to-day that you forget to think about how it is, sometimes, for the person, and just thinking about it now, I do wonder what must it be like in there? Is it confusing? Or does he not realise? You can’t comprehend it, can you? Losing the structure of who you are. (Abbie).

The majority had a sense of having difficulty interacting effectively and appropriately with the person they cared for, due to cognitive, memory, and communication problems. These ranged from minor difficulties with everyday conversation and memory to profound inability to communicate. Many reported frustrations with their relative’s memory problems and the resulting repetition of questions, conversations, and behavior. Such problems could result in distressing arguments and conflict between the carer and the person cared for. In rarer cases, carers also had to cope with violence. Carers sometimes found it difficult when their relative had said or done something upsetting toward them and then forgotten the incident, as this highlighted the loss of relationship and their degree of isolation. All these difficulties could result in carers feeling their relative no longer cared about them, even if they knew this was a result of dementia-related difficulties.

Yeah, he’s forgotten, yeah, and he can get violent sometimes, and he has hurt me, but he’s forgotten, and he’ll say, “What are those bruises?” And I’ll say, “You did it,” and then he gets so upset, but [sighs] what can you do? (Annabelle).

Many felt their role in the relationship had become like that of a parent. This affected relationship dynamics differently for spouses and adult-children but, in each case, presented challenges and required adaptation. In this context, many said it was difficult to balance dementia-related care needs with respecting the feelings and wishes of their relative. Knowing when to correct or help their relative and when to leave their relative to do things independently was a challenge that often resulted in uncomfortable feelings of uncertainty and guilt. Several carers described how distressing it could be to have to upset their relative in order to do “the right thing” for them, for example, persuading the relative to attend respite or day care or to receive personal care. Some found it particularly distressing if they had to “trick” their relative into compliance:

It becomes the norm and I hate the trickery to get her to the homes, I don’t like lying to her. See they come with coaches for her, if she won’t get on the coach I have to take her in the car so then I have trick her, I say “Come on we’ll go to Morrison’s [shop]”, and I take her to the Home . . . .I know full well she’s beginning to realise that I’m tricking her, you know, and you don’t like lying to a person you love, you don’t, I just don’t like doing it. (Evan).

As part of trying to feel close, carers discussed wanting to provide their relative with the best possible quality of life. As they could no longer share previous activities, carers often made efforts to find new ways to connect, for example, through joint outings, such as attending activities/groups or walking, or indoor activities such as sharing music, reading, or simply sitting together: “Um, when we’re just sat watching a film of whatever, you know, she sings to it and usually and she starts singing of something, so that’s quite nice really.” (Charlotte). Some carers tried to ensure their relative’s friends and family spent time with them so they still had social interactions.

It was a challenge for carers to maintain a secure attachment to the person they cared for. Carers knew the dementia would have an increasing impact and that the person cared for would ultimately not survive. Some found it was difficult to enjoy time with their relative because they felt so acutely aware these times would not last: “I have awful thoughts thinking, ‘Well, I wish it had been a brain tumour’, because they could either have treated it or it would have been …..quicker.” (Abbie).

#### Feel in Control of my Life

All carers discussed, explicitly and implicitly, the need to have a sense of stability and security through feeling in control of their own lives, having control over what was happening around them or being able to plan and execute plans. The vast majority said the uncertainty of how and when symptoms would manifest and progress was destabilizing as they were continually having to adjust to new issues. One wife said: “I think if you look too far into the future you frighten yourself and wonder how you will cope, and I genuinely don’t think too far ahead.” (Theresa). Many found this uncomfortable, difficult, and distressing, although many also said they had been able to adjust to things they would have thought impossible. One daughter spoke of learning to accept things as they occurred: “But I have had to realise that I’ve got to be so flexible and not get upset about that.” (Susan).

In addition, although many said they felt compelled to accept some help from others in order to continue their role, this often undermined their sense of control over their own life and that of their relative. Making decisions about who provided care, how, and when was very important. Many carers felt they knew, or had learnt, the most successful and appropriate ways of meeting the needs of their relative, as when Theresa stated: “I just thought nobody could do it like I could, I think.” This was coupled with feeling that, as they cared about the person more than others, others would not be as attentive to their relative’s needs. The care provided by others was frequently outside the carer’s control and often fell short of his or her expectations. Some perceived that accepting help caused more problems than it solved because changes to routine could destabilize the person they cared for, causing anxiety beforehand and distress afterward. Therefore, it was very important to carers that support was timely and appropriate, yet often support services were not adequately tailored to the specific needs of their relative and/or did not arrive at a useful time.

She became incontinent, that was a major problem as far as I was concerned, and eventually I had to arrange for carers to call. Carers have been coming three times a day, which is helpful in a way but the fact that they only spend a maximum of fifteen minutes, which is like in effect that’s 45 minutes out of 24 hours, it’s a marginal benefit really because she doesn’t operate her bowels according to when they come. (David).

The quality of information and support could significantly impact on the degree to which carers felt in control. Carers needed access to support and information about how to provide care, finances, medical issues, future plans, and social care at the right time for them. Some felt they had been left to learn how to care on their own through trial and error. It seemed important that support from these sources was genuinely caring and nonjudgmental because carers often described feeling unsettled when having to deal with different opinions or judgements about their approach to care, as in this quotation in which Geeta feels criticized: “Well, sometimes it’s annoying, because whatever I do, that stays on one side, and what I haven’t done, they point that stuff out.... ‘Why have you got a sink full of dishes today?’, all that stuff.”

Overall, it seemed carers were more able to feel in control when they had a combination of support, from health professionals, people with understanding of caring (e.g., other carers), and friends/family.

#### Freedom

All carers described a need to have time to themselves, to relax, to attend to their responsibilities, and to be spontaneous. The provision of instrumental support and the need to monitor their relative’s safety restricted carers’ freedom, with this varying across the sample depending upon how much assistance and supervision was required. Many carers described the constricting impact of routines and the length of time needed to accomplish tasks. Routines were sometimes in place to anchor the relative with dementia in a predictable framework and sometimes self-imposed as a way of managing the provision of care. Some felt their constant presence helped their relative feel anchored, but this was clearly a restriction on the carer’s freedom, as in this statement: “I feel I’m attached on a piece of string to my husband and that string is getting shorter and shorter and shorter.” (Beatrice)

To generate free time, some carers called on support from friends, family, or formal services. However, despite knowing their free time was limited, the majority described how difficult it was to relax or “switch off” because of anxiety about the quality of care being provided, the relative’s safety, and feeling guilty about being away. Some said completing practical tasks, such as shopping or paperwork, filled all the free time they were able to generate, resulting in very limited time to relax or pursue social or leisure activities: *“*It’s like you’re on call 24–7” (Jasmine).

Caring for someone with dementia also restricted people geographically, with many saying they did not travel far because they feared a crisis might arise and they would feel guilty if they were not available: “It sort of, cuts into every day basically, you know, so we can’t really do anything. If we wanted to do something, you know, we’re stuck.” (Felix).

#### Feel Connected to the People Around Me

All carers described the way caring for their relative with dementia impacted on other relationships, reducing their networks and leading to feelings of isolation. Caring responsibilities reduced the amount of time and energy carers had to spend with others, and this was often further compromised by perceived difficulties with sharing thoughts and feelings with others and anxiety about how their relative with dementia might behave in public. Therefore, carers were less likely to socialize, with or without their relative present. When carers maintained social contacts, these appeared to provide an important area of well-being and connectedness: “My friends have, yeah, yeah, yeah, they’re my medicine, my friends.” (Beatrice)

As well as needing to feel connected to others, carers also discussed the importance of friends and family helping to provide care. When friends and/or family were sensitive to the carer’s needs, this could be an important facilitator for feeling connected, and some also said they had made valued connections with other carers. However, some felt other people added to their stress or distress: “They don’t know how to approach Mum and then Mum can’t accept the way they are because all they’ve got is a list of complaints.” (Jasmine). Many said it was enough for the network surrounding them simply not to cause any additional difficulties or distress.

Carers said caring dominated their attention and energy, loosening connections with others, as they often had little left to put into other relationships.

#### Protect My Relative

The need to protect their relative was present in the accounts from almost all carers. This seemed to come from an underlying instinct to protect close vulnerable family members.

They can drive you absolutely crackers! They can more or less ruin your own life and you have to give up so much but…when I see her in those moments when she’s like a child or when she’s lost and she’s looking to me to guide her, just those few moments then everything becomes secondary and I just think, I’m gonna help you and that’s all there is to it. (Karl).

Carers discussed wanting to ensure the physical safety of their relative, including their ability to successfully negotiate the home environment and the possibility they would get lost when out and about. In public, they made efforts to protect the relative’s dignity and many carers developed ways to protect their relative and, in some cases, also to protect the general public.

I feel hugely embarrassed, and I know I shouldn’t, and I know I should cope with it, but it’s very difficult to cope with, and I read a thing only last week on the Alzheimer’s Forum, they have a chat forum. Somebody had come up with this brilliant idea, which I think I will use, of making little cards with just a little explanation on to just quietly pass to someone in the street or in a restaurant or in any situation where you were embarrassed because they were doing what they do, and people were not coping with it very well. (Beatrice).

#### Take Care of Myself

For the majority, caring for somebody with dementia negatively impacted upon the need to maintain their own health. Carers described how their emotional resilience and stability had become eroded over time because of the cumulative effect of the many facets of caring. Many carers said they always felt tense or anxious and were not in control of their emotions to the same extent as before they began caring.

The combination of their own anxieties and the disorientated behavior of their relative with dementia often resulted in carers having disturbed sleep, which could further erode their resilience. Despite several carers discussing anxiety about what would happen if they were no longer fit enough to provide care, many found it difficult to find time for and prioritize their own health needs.

I’ve not really focused on my own physical, well physical and mental health needs so, I find that, you know, health problems have cropped up all of a sudden, that needed my attention but I was, you know, not paying attention on that side and focusing on my mum. And plus, physically tiring because…I wasn’t really getting a proper sleep or going into my bed…it’s affecting work, you’re at work, I’m feeling really, really exhausted and tired. So it’s all, you know, because of the extra things I have to do, it’s sort of impacted on me, you know. So tired. (Jayda).

#### Be My Own Person

Most carers discussed the need to maintain or develop their sense of themselves. To enable this, carers needed to feel true to their values, be able to attend to the important aspects of life, feel they were moving forward, and have good self-esteem. In rare cases, carers found that caring had provided them with a sense of purpose and enhanced their self-esteem; others felt caring had not changed them as a person, but for the majority caring was detrimental to their sense of being their own person:

Well I know my daughter asked me about relationships, she said, “Would you get married, mum or, you know, have a relationship,” and I say to her, “Oh, to be honest, I don’t have even, I don’t think I have time for such things in my life, because I’m so tired looking after mum and dealing with all that that I just couldn’t. (Jayda).

Many said they had put their own life on hold “And things that I wanted to do, would have wanted to do got put on the backburner and I feel as though this has become so central.” (Alfred). Others felt that life was now moving in a direction they had not desired.

The emotional and physical demands of caring often made it difficult to fulfil aspects of life fundamental to identity (e.g., work, hobbies, family, and social roles), and when they did have opportunities to attend to these areas, carers often felt guilty about doing something for themselves. Some carers experienced guilt, but also realized the importance of maintaining their own interests.

I would go mad if I wasn’t working. I think you have to, I think if you can maintain some normality, you’re also looking after yourself, I suppose it’s like putting your life jacket on first isn’t it before you deal with your child. (Charlotte).

It appeared difficult for many carers to feel good about themselves and the care they provided. Carers often had layers of guilt about not providing good enough care; not being patient, resilient, or caring enough; not providing good quality of life for their relative; not fulfiling their other roles well enough and having time for themselves. Caring for someone with dementia also challenged carers’ sense of integrity if they had to do things that they were not comfortable with, such as using deception with their relative.

#### Share/Express My Thoughts and Feelings

Most carers discussed the dynamics of sharing their thoughts and feelings. Some were comfortable to keep their feelings to themselves but the majority discussed the need for some degree of sharing with others. Some described having good outlets through friends, family, or other carers, but for many, this was complex in terms of how and when to express themselves, what to share, and who to share with.

Some carers felt all their conversations had become dominated by dementia and caring, but a lot of the content was superficial, practical, or judgemental. Carers often said other people could not understand what it was truly like to be a carer unless they had experienced it themselves, and in some cases, this meant carers did not think it was worthwhile sharing. The perception that others were not genuinely interested or would be burdened by their disclosures also reduced some carers’ willingness to share.

Some worried that revealing negative feelings or thoughts about caring might lead to unwanted interventions (e.g., the relative with dementia might be taken away from them) or were concerned about friends or family judging them and drawing wrong conclusions.

It’s almost a self-protection thing, isn’t it? Maybe it’s just me, some things you just keep, keep closed because it’s, maybe you make yourself vulnerable to, if you say something in an unguarded moment, you can’t take it back………they’re creating their own little narrative about what’s going on, I don’t know. I just feel a little bit like I need to keep a little space. (Abbie).

#### Get Things Done

Two-thirds of carers described frustration arising from difficulties in being able to address necessary things in their life. This ranged from the time and effort needed to complete basic care tasks, through to more generalized feelings of lacking energy for their own life plans and difficulties maintaining or developing work, social life, and other relationships. It could generate an overwhelming feeling of never being on top of things within their care role or their life in general.

The logistics of organizing support and care often involved multiple agencies, professionals, friends and family, and carers found that bureaucracy added to an already frustrating and time-consuming process. The reduced capacity of the person they cared for, the desire to ensure good quality care, and the need to get the right support at the right time resulted in a lot of carers feeling they needed to coordinate arrangements themselves. A number of carers described moments when they became overwhelmed: “Some days all those plates come crashing down at the same time, ’cos the plates stopped spinning. Fortunately it’s rare, but it can happen and when it does happen then, yeah, I have a meltdown.” (Isobel).

## Discussion and Implications

We have presented an inductively derived needs-led framework that aims to capture how providing care for a relative with dementia impacts upon carers’ ability to fulfill their own needs. While carers were given opportunities to talk about how care had enhanced their fulfillment and some gains were mentioned, the accounts were predominantly focused upon the ways that caring for their relative with dementia restricted and eroded the carers’ sense of being able to fulfil their needs. The most pervasive themes related to the way dementia impacted on carers’ ability to continue to feel close to their relative, even though they wished to do so; the ability to feel in control of day-to-day life and the future; their ability to fulfill their needs to have time and space for relaxation or spontaneity; and their need to feel connected to others.

To consider the plausibility of the needs we have identified, we compared our framework with other needs-led theories and evidence. In doing so, we first make the point that research evidence supports the range of needs identified by Maslow but not their hierarchical nature (Tay & Diener, 2011). We therefore compare carers’ needs with those of other populations but without placing them in Maslow’s hierarchy. Table 3 maps the needs impacted by caring against those identified in four other frameworks. In all cases, we have attempted to identify the closest matches in meaning against the themes in our framework.

**Table 3.** Comparison of the Needs-led Carers’ Framework with Needs Identified in Other Populations and Frameworks

Human needs (Maslow, 1943)	Dimensions of well-being (Ryff, 1989)	Carer’s needs (Current framework)	Needs of people with dementia (Schölzel-Dorenbos et al., 2010)	Needs impacted by Crohn’s disease (Wilburn et al., 2015)
Belongingness and love	Positive relations with others	Feel close to my relative	Affection, love, and acceptance	Affection
Belongingness and love	Positive relations with others	Feel connected to the people around me	Social roles	Social needs
Belongingness and love	Positive relations with others	Share/express my thoughts and feelings	Affection, love, and acceptance	Social needs
Safety	Positive relations with others	Protect my relative	Safety—prevention of harm	Safety and security
Self-actualization	Self acceptance/ personal growth	Be my own person	‘Being’ need	
Self-actualization	Autonomy	Freedom		Autonomy
Esteem	Environmental mastery	Feel in control	Mastery and independence	Autonomy
	Environmental mastery	Get things done	Mastery and independence	Autonomy
Biological and physical		Take care of myself	Maintaining personal hygiene, housing and feeding	Physical needs

*Note:* An empty cell indicates lack of an equivalent theme.

In comparing the set of needs we have identified against universal human needs proposed by Maslow (1943) and Ryff (1989), it is apparent that dementia caregiving appears to impact the whole range of needs in each of their frameworks. A needs-led study of how Crohn’s disease impacted on fulfilment of needs (Wilburn, McKenna, Twiss, Kemp, & Campbell, 2015) is the only one of the four needs-based frameworks mentioned here that was derived inductively from interviews with participants. Our own findings connect well with their relational categories (affection and social) and with their identified need for autonomy. Overall, these comparisons provide some plausibility to our findings, in showing considerable overlap alongside some variation that can be hypothesized as being due to the different population we have studied.

It is striking that relational needs are prominent in our analysis. Our framework identified four variations of needs in this area, captured under the need for positive relations with others in Ryff’s framework. Three of these also appear to be connected with needs for belongingness and love according to Maslow’s framework. It seems intuitively correct that caring for a relative to whom one has been attached, who is now becoming progressively cognitively impaired, would impact on the relationship, and indeed this has been noted in a range of studies (Ablitt, Jones, & Muers, 2009; Quinn, Clare, & Woods, 2009; Riley, Evans, & Oyebode, 2016).

The proposed hierarchy of needs of people with dementia by Schölzel-Dorenbos et al. (2010) can be considered alongside the needs in our framework from the point of view of reciprocity between those living with dementia and their carers. Our findings would appear to lend additional validity to Schölzel-Dorenbos’ conclusion that unmet needs for relational fulfilment in both partners add to carers’ sense of burden. For example, just as we found that carers’ needs for closeness with their relative were impacted by dementia, so Schölzel-Dorenbos et al. found that people living with dementia have a strong need for love, affection, and acceptance. Similarly, carers’ need for sense of connection with those around them is echoed by Schölzel-Dorenbos et al.’s identified need for people with dementia to retain social roles. A further pairing of needs, between our framework and Schölzel-Dorenbos et al., links carers’ need to try and protect their relative both physically and psychologically with the person with dementia’s need for safety and prevention of harm.

In comparison with stress-appraisal-coping models of caregiving, a number of the needs impacted upon by dementia care that are included in our framework illustrate stresses that are well known to be associated with caring, including those that impact upon a sense of control, time for self-care, and freedom to live life as one might like. These echo narratives of stress, burden, role captivity, and entrapment (see e.g., Pearlin et al., 1990). One advantage of the needs-led framework may be that it complements stress-appraisal-coping models by revealing one mechanism through which stressors associated with dementia care may have an impact on the morale and well-being of the carer. We propose that the impact of stress may relate to the way stressors affect carers’ ability to fulfil human needs, a hypothesis that could be empirically tested. A further key advantage of the needs-led approach is capacity to reveal needs-fulfilment derived from caring (such as enhancing a sense of purpose). This provides a connection between the needs-led approach and research on positive aspects of caring. Further research to consider satisfactions and positive growth from a needs-led perspective would broaden further our understanding of how caring impacts on fulfilment of human needs.

In reflecting upon the emergent themes, we have considered whether they reveal any aspects unique to dementia caring. Many of the examples from our participants highlighted the way changes in awareness, memory, and capacity of the person cared-for impeded needs-fulfilment in multiple domains. It may be that these are aspects that make fulfilment of needs more challenging for carers of people with dementia than for those caring for a relative with another condition.

Our study has a number of limitations. We found it hard to recruit young carers, daughters-in-law, and male South Asian carers. We were pleased, however, that some who are often omitted from carer research did participate, including people from more remote rural areas, from South Asian and black communities, a young carer, and a number of sons. We cannot claim that our nine themes are definitive as they emerge from our subjective understandings. However, as they were drawn inductively from those caring for a relative with dementia, they may hold validity and credibility for others in a similar position. Although we aimed to uncover underlying needs through pursuing Socratic questioning, at times we interpreted statements rather than underlying needs having been stated explicitly by the participant. On the other hand, the interviewers were new to work in this field, which freed them to interview without theoretical preconceptions, facilitating the inductive approach.

One strength of a needs-led account is its capacity to show what underlies some of the struggles carers may experience in providing dementia care. The needs that have emerged from our inductive analysis have implications for policy and practice, which could help to sustain family care. They highlight the potential benefit of supporting carers through focusing on enabling them to continue to fulfil needs as they embark on caring, rather than targeting stress reduction or knowledge per se. It is therefore vital for dementia strategies to consider the person with dementia in the context of the dyad or family, not as a “clinical case” in isolation. An explicit discussion around the nine needs could be a starting point in considering life beyond diagnosis for family members who are likely to become carers. This could lead into a consideration of which needs are important to particular individuals or dyads and to the formulation of plans to ensure the impact of dementia on these needs is minimized. In the United Kingdom, this could be incorporated into the formal Carer’s Assessment to which all carers are entitled (Care Act, 2014). In addition, although not the primary focus in this study, the needs-led approach has potential to yield valuable information about ways carers manage to address their needs despite the constraints of caring.

Emphasis on a relational approach and on retaining a sense of mastery over the situation could be particularly helpful. The centrality of the themes around relationships in our needs-led framework strongly implies that clinical services need to pay attention to enabling dyads, where one has dementia and the other is providing care, to maintain a positive relationship. Programmes such as SHARE (Support, Health, Activities, Resources & Education; Whitlatch et al., 2015), for example, may help to minimize the impact of dementia on fulfillment of this need.

In this paper, we have reported on the initial inductive phase of an ongoing research study to develop a measure of quality of life for carers of people living with dementia. Our analysis highlighted nine fundamental needs that are affected when caring for a relative with dementia. These needs were widely endorsed across a sample of diverse carers and have provided the building blocks for the development of a needs-led carer quality of life tool, which is currently being evaluated. The needs-led framework offers a perspective that captures stress, burden, and loss associated with caregiving but which can also reflect the positives that the experience of caring may offer.

## Supplementary Material

Supplementary data are available at *The Gerontologist* online.

## Funding

This work was supported by the Medical Research Council and the National Institute for Health Research (NIHR). Grant title: HQLC Dementia Carers Instrument Development: DECIDE (MR/M025179/1). Chief Investigator: Dr Penny Wright.

## Supplementary Material

Supplementary File 1
