# Supplementary material for: A Needs-led Framework for Understanding the Impact of Caring for a Family Member With Dementia
Source: Gerontologist. 2017 Oct 9;58(2):e68–77. doi: 10.1093/geront/gnx148 (PMC5946854; doi:10.1093/geront/gnx148)
Supplement: Supplementary File 1 [file gnx148_suppl_supplementary_file_1.docx]

Supplementary file 1

**Interview Guide**

Can you tell me a bit about who it is you care for and how this came about?

What do you like and dislike about providing care?

How is care positively and negatively affecting your relationship with X?

What do you think X feels about you having to care for them?

What are the best times between you and X?

Since you first started to provide care, how has it positively and negatively affected your life?

How is care positively and negatively affecting your health?

How is care positively and negatively affecting the way you think about yourself?

What do you think other people think about you being a carer?

What do you think the future holds for you?

What new things have you learnt through needing to provide care?

Thank you and summarise what the researcher has understood, with a few key points.

Is there anything else that you would like to add that we have not talked about so far?

How have you found the interview? Do you have any advice for my future interviews?

**Probes**

Follow up responses to uncover underlying needs impacted by caring. Apply successive probes to the same area until the interviewee is unable or chooses not to disclose any more depth of understanding.

So why do think this situation made you feel this way?

So what was it about that incident that made you think this?
